# Natural Polysaccharides and Their Derivates: A Promising Natural Adjuvant for Tumor Immunotherapy

**DOI:** 10.3389/fphar.2021.621813

**Published:** 2021-04-14

**Authors:** Ye Li, Xiaomin Wang, Xiaoran Ma, Cun Liu, Jibiao Wu, Changgang Sun

**Affiliations:** ^1^ College of First Clinical Medicine, Shandong University of Traditional Chinese Medicine, Jinan, China; ^2^ College of Traditional Chinese Medicine, Shandong University of Traditional Chinese Medicine, Jinan, China; ^3^ Qingdao Academy of Chinese Medical Sciences, Shandong University of Traditional Chinese Medicine, Qingdao, China; ^4^ Department of Oncology, Weifang Traditional Chinese Hospital, Weifang, China

**Keywords:** natural polysaccharides, tumor immunotherapy, antigen presenting cells, natural killer cells, natural adjuvant

## Abstract

The treatment process of tumor is advanced with the development of immunotherapy. In clinical experience, immunotherapy has achieved very significant results. However, the application of immunotherapy is limited by a variety of immune microenvironment. For a long time in the past, polysaccharides such as lentinan and *Ganoderma lucidum* glycopeptide have been used in clinic as adjuvant drugs to widely improve the immunity of the body. However, their mechanism in tumor immunotherapy has not been deeply discussed. Studies have shown that natural polysaccharides can stimulate innate immunity by activating upstream immune cells so as to regulate adaptive immune pathways such as T cells and improve the effect of immunotherapy, suggesting that polysaccharides also have a promising future in cancer therapy. This review systematically discusses that polysaccharides can directly or indirectly activate macrophages, dendritic cells, natural killer cells etc., binding to their surface receptors, inducing PI3K/Akt, mitogen-activated protein kinase, Notch and other pathways, promote their proliferation and differentiation, increasing the secretion of cytokines, and improve the state of immune suppression. These results provide relevant basis for guiding polysaccharide to be used as adjuvants of cancer immunotherapy.

## Introduction

Cancer remains one of the major health threats worldwide despite the continuous development of diagnostic tools and therapeutic drugs. Updated immunotherapy plays a role in the treatment of tumors, especially the immunotherapy represented by immune checkpoint inhibitor (ICI) gradually shows significant efficacy. Despite numerous reports of clinical benefits from immunotherapy, real-world studies have shown a low response rate to immunotherapy, with fewer than 20% people responding (Haslam and Prasad, 2019). This pessimistic response rate may be closely related to the different microenvironmental states of immune inhibition in tumor patients.

**FIGURE 1:**
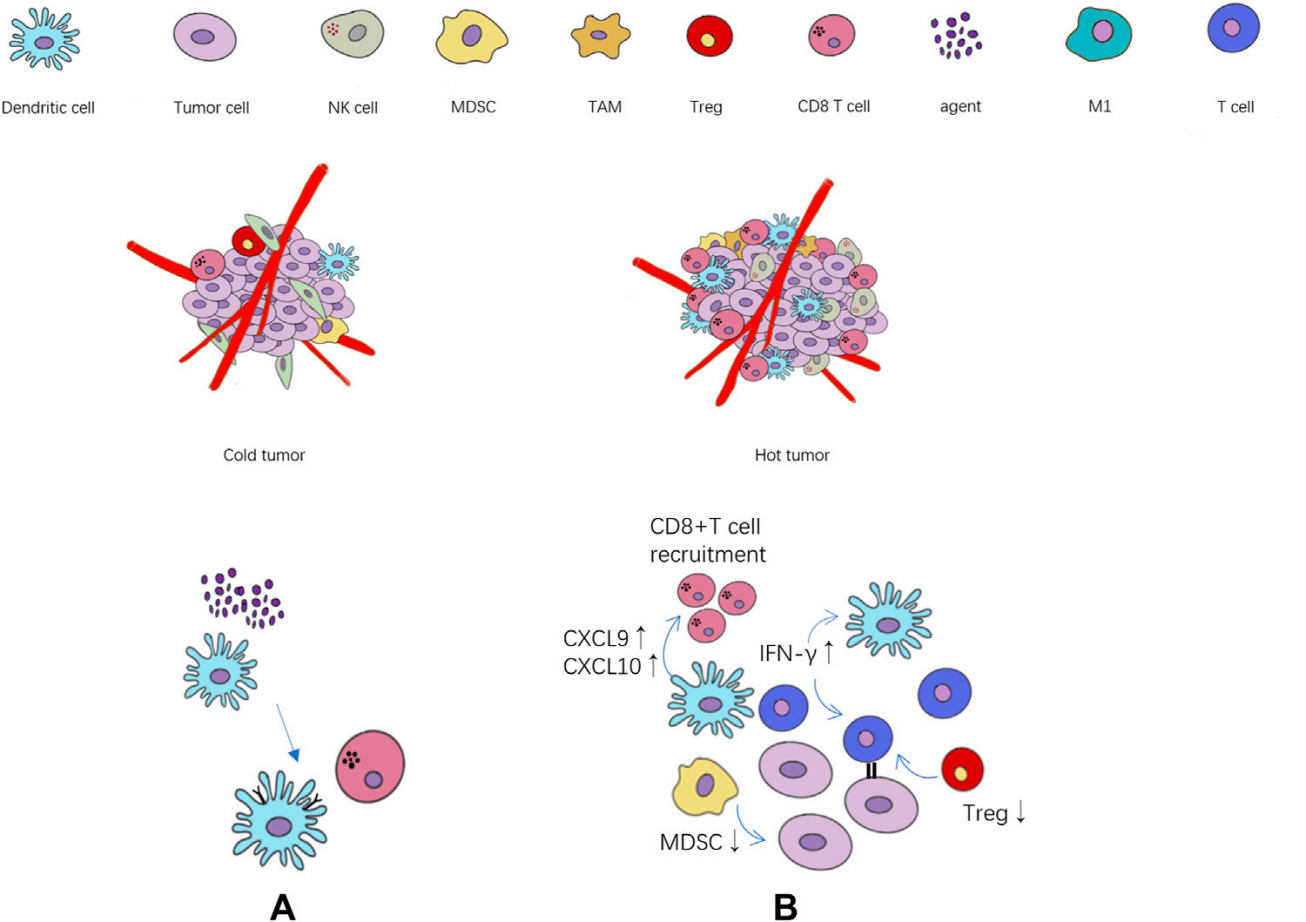
The relationship between the state of tumor immune cell infiltration and immunotherapy. **(A)** The agents activate the maturation of dendritic cells, and further activate CD8+ T cell. **(B)** Cytokines, chemokines and immune cells associated with immune activation and immunosuppression state. ↑: immune activation ↓: immune suppression.

The state of immune microenvironment is crucial to immune efficacy, and immune cytokines play a key role in the transformation of immune microenvironment by regulating immune cells. In the tumor immune microenvironment of ineffective population (cold tumor), the immune infiltrating cells are often in a state of lack or inhibition, and the tumor itself will also produce immunosuppressive cytokines (Duan et al., 2020), further exacerbating the immunosuppressive state. On the contrary, the effective population (“hot” tumor) are characterized by the accumulation of pro-inflammatory cytokines and immune cells infiltration. The activation of immune effector cells promotes the production of cytokines and specific antibodies, which can better trigger the immune response (Figure 1). A research team at the University of North Carolina (Hollern et al., 2019) found that in triple-negative breast cancer, T follicular helper cells activated by ICIs regulate the production of antibodies by B cells, which are critical to the efficacy of immunotherapy. In addition, natural killer cells (NKs) can lyse tumor cells by recognizing tumor-derived antigens or cell surface stress molecules (Paul and Lal, 2017). Macrophages can directly participate in tumoricidal activity through antibody-dependent cell-mediated cytotoxicity (ADCC) and antibody-dependent cellular phagocytosis (ADCP) (Ginefra et al., 2020).

Immune cells are divided into innate immune cells and acquired immune cells. In the course of tumorigenesis and development, the innate and adaptive immune systems play a role in host protection. Immune cells infiltrate tumors, co-evolve and cooperate with tumor cells, create a microenvironment of inflammation and immunosuppression, and promote tumor growth and spread. The innate immune system can directly inhibit tumor progression by participating in the tumor-killing activity, or it can indirectly participate in the anti-tumor process by releasing various inflammatory cytokines and recruiting adaptive immune cells (Fridman et al., 2017; Ginefra et al., 2020).

When the solid tumor grows to a certain extent, it will be invasive and cause slight damages in the surrounding tissue, inducing inflammatory signals, thus causing innate immune cells to recruit to this site and stimulate immune cells to produce IFN-γ and various chemokines (Tang et al., 2012), which stimulate the innate immunity, causing the death of tumor cells. The tumor cell fragments formed after apoptosis are absorbed by local dendritic cells and migrated to the draining lymph nodes, thus inducing tumor-specific CD4+ and CD8+T cells to migrate to the tumor site, producing the acquired immunity and stimulating the anti-tumor activity.

Innate immune cells (macrophages, neutrophils, DCs, and NKs), in conjunction with adaptive immunity (T and B lymphocytes), provide a strong first line of defense against cancer cells, detecting and eliminating more immunogenic cancer cells and counteracting spontaneous tumor growth (Pasto et al., 2020). Adaptive immunity is not an autonomous process, requiring antigen presentation from specialized cells in a pro-inflammatory environment. It is undeniable that the application of T cell immunotherapy in cancer treatment has achieved unprecedented success. However, its application is still limited to several tumor types. In this context, the regulation of innate immunity seems to have important implications for the intervention of tumor immunotherapeutic effects. Therefore, it is worth exploring whether innate immunity can be used as a potential target for immunotherapy or whether adaptive immunity can be further regulated through the activation of innate immunity.

It has been found that the innate immunity can have an impact on current T cell cancer immunotherapy and can provide potential opportunities for the development of new therapeutic strategies. Therefore, we need to look for drugs that regulate the non-specific immunity. At the same time, natural compounds have attracted people’s attention because of their wide range of non-specific targets and high level of safety. Polysaccharides, polyphenols, flavonoids, terpenes, saponins, and other natural substances have been proved to have anti-tumor and immunomodulatory effects (Deng et al., 2020). However, we believe that compared with other drugs, natural polysaccharides are biological response modulators (BRM), which produce a wide range of immune enhancement mainly by activating the host immune system, including the innate and acquired immunity (Jiang et al., 2010; Hou et al., 2020). It has little cytotoxicity in humans and has great potential for combination therapy. Additionally, their extraction source drugs have better immune regulation effects on the body, and are more suitable for the construction of follow-up derivatives. In the clinical experience, their extracted source drugs have better immune regulation ability and anti-tumor activity on human body, and the diversity of composition and structure is more suitable for the construction of subsequent derivatives. Furthermore, a number of experimental studies have shown that natural polysaccharides can regulate the host immune system and activate the anti-tumor activity of immune cells in a tumor microenvironment (TME). Therefore, the study of natural polysaccharides on tumor immunotherapy is of great significance. This review discusses the regulation effect of innate immune regulation on tumor immunotherapy and natural product polysaccharide on innate immune cells.

## The Regulatory Mechanism of the Tumor Immune System

### The Immunomodulatory Mechanism of Macrophage Cell

Macrophages, one of the important members of the body’s innate immune defense Frontier, derived from bone marrow progenitor cells (Hume et al., 2002), playing an important role as a bridge between the innate immunity and acquired immunity. They exist in almost all tissues and their main functions are phagocytosis and degradation of dead cells, cell fragments, and foreign pathogens, as well as coordinating inflammatory processes (Hume, 2006; Bonnardel and Guilliams, 2018). Furthermore, macrophages can present antigens, secrete a variety of active substances, as well as regulate the local microenvironment and other physiological functions (Gordon and Plüddemann, 2017).

Macrophages can be activated by different stimuli to polarize M1 and M2 phenotypes, which reflect the characteristics of Th1 and Th2 cells, respectively (Locati et al., 2013). CD86 is considered to be a marker of M1 macrophages, while CD206 is usually used to identify and screen M2 macrophages (Shapouri-Moghaddam et al., 2018; Wang et al., 2019). Under the stimulation of IFN-γ, microbial stimulation (such as LPS), and cytokines (TNF, GM-CSF), macrophages often polarize to the M1 phenotype, with high bactericidal, bacteriostatic, pro-inflammatory and tumor cytotoxic activity, and the potential to kill tumor cells. However, in the TME, differentiated macrophages are typically polarized to the M2 phenotype by anti-inflammatory molecules (such as IL4, IL-13, etc.) (Mantovani et al., 2004; Locati et al., 2013; Tao et al., 2020). Type M2 tumor-associated macrophage (TAM) contributes to tissue repair, tumor angiogenesis, wound healing, and promotes the survival, proliferation, and spread of tumor cells (Wei et al., 2019; Tao et al., 2020). Therefore, in order to balance the level of tumor immunosuppression and immune activation for killing or clearing the tumor, we need to polarize macrophages to M1 or adjust the ratio of M1 to M2.

As a group of cells characterized with plasticity and pluripotency, macrophages show significant functional differences under the influence of different microenvironments. TAMs exist widely in the anoxic region of TME, and most of them are M2 polarized, resulting in immunosuppressive phenotype and inhibition of T cell-mediated adaptive immunity. A series of studies have shown that TAMs affect the therapeutic effect of anti-pd-L1 and anti-CTLA-4 drugs to a great extent, and the curative effect has been observed when traditional immunotherapy were combined with macrophage targeting strategy (Ginefra et al., 2020), but its specific mechanism needs to be further explored.

In general, the recruitment, phagocytosis, survival and functional of macrophages show a high correlation with specific immunity, while the polarization and distribution of M1 and M2 macrophages have been shown to affect the course and treatment response of cancer (Larionova et al., 2020). Therefore, the adjuvant therapy strategy of macrophages as drug targets may improve the efficiency of immunotherapy in an all-round way.

### The Immunomodulatory Mechanism of Dendritic Cells

The dendritic cells (DCs) are a subset of innate immune cells that are the key mediators of anti-tumor immunity, while T lymphocytes are the key components of anti-virus and anti-tumor immunity. Further elucidating the biological mechanism of DC infiltration and activation in tumor and controlling T cell immunity will be of great significance for the selection of a reasonable combination therapy in the future. Conceptually, this will promote the transformation of T cell inflammation, and increase the proportion of patients who benefit from cancer immunotherapy (Garris and Luke, 2020).

Dendritic cells are generally divided into plasmacytoid precursor DCs (pDCs) and conventional DCs (cDCs). In previous studies, pDC is mainly by producing I-IFN to participate in antiviral immunity or other dendritic cell activation; In contrast, cDC can further differentiate through the expression of surface immune receptors, or through cross-present antigens and other ways to activate anti-tumor specific immune effects (Chan and Housseau, 2008). Thus, the DC can be used to stimulate immature T lymphocytes, which forms a heterogeneous group of specialized antigens presenting cell (APC), and its function is integrated into innate and adaptive immune responses (Ginefra et al., 2020).

Tumor cells often create an immunosuppressive microenvironment through some mechanisms, affecting the maturation and activation of DC, leading to insufficient T cell activation, and possibly inducing T cell tolerance to tumor-associated antigens. In addition, metabolites in TME (such as lactic acid, etc.) also inhibit the function of DC (Wculek et al., 2020). As the APC in tumor microenvironment (TME), the DC initiates the cancer immune cycle by cross-presenting tumor-associated antigens to naive T cells (Ginefra et al., 2020). VEGF, IL-6 and IL-10, which are usually overexpressed in TME, can activate STAT3 signaling, thereby inducing an immature tolerance phenotype in tumor-associated DCs and promoting tumor progression (Nefedova et al., 2004; Yu et al., 2009). The regulation method mentioned in this article is a general summary of the experiment of plant polysaccharides to activate DCs. In the past, people have tried a variety of polysaccharides to regulate DCs, thereby improving the body’s anti-tumor immunity.

### The Immune Regulation Mechanism of Natural Killer Cells

NK cells are important innate immune cells that constitute the first line of defense against microbial infections and cancer development, with a strong killing function against tumor cells, virus-infected cells, and other physiologically stressed cells (Paul and Lal, 2017; Trefny et al., 2020). The NK cell activity is a significant index of the non-specific immune system. They can not only directly kill tumor cells by secreting cytoplasmic particles, releasing various cytokines, participating in death receptor-mediated apoptosis and ADCC, but also indirectly play a role by producing different cytokines and chemokines interacting with other immune cells (Trefny et al., 2020).

NK cells promote the recruitment of DC to solid tumors by releasing a variety of chemokine ligands (such as CCL5, XCL2, etc.), and promote the polarization of Th1 cells by releasing IFN-γ (Chiossone et al., 2018). In TME, DC-NK crosstalk can promote the maturation of DC, which makes DC secreting IL-12 and promoting the expression of CD86 so that to enhance the activation of CD8 T cell. NK cells can also indirectly regulate T cells by regulating APCs to ensure the initiation of T cells (Pierce et al., 2020). Therefore, there is a significant correlation between tumor-related immune function impairment and the biological function of NK cells.

In cancer patients receiving various treatments, the immune function may be reduced or impaired, resulting in decreased cytotoxic T lymphocytes and NK activity. For advanced cancer patients, NK activity is significantly reduced and cytokine production is impaired and related to poor prognosis (Gao et al., 2005a). When some NK cells did not express ligands for MHC-I molecules, or when there was a lack of MHC-I presentation, they could be regarded as hyporesponsiveness of NK cells (Zimmer et al., 1998; Anfossi et al., 2006). Additionally, the expression of PD-1 on NK cells may be related to the low responsiveness of NK cells (Trefny et al., 2020). Therefore, the low reactivity of NK cells may be related to the clinical benefits of ICIs. Regulating the activity of NK cells can synergistically regulate multi-level immune response, and finally achieve protective and lasting immunity against tumors.

In short, tumor immunotherapy needs to activate the immune mechanism again and maintain its dynamic balance. However, according to the heterogeneity of individuals and tumors, the effects of immunotherapy are very different: non-synonymous somatic mutations in individual genes or low genomic mutation load are related to the lack of clinical benefits and immune resistance of ICIs (Law et al., 2020). The expansion of immunosuppressive cells, the expression of inhibitory cytokines and proteins, as well as angiogenesis suppress the immune response, which is the reason for the unsatisfactory clinical results of cancer vaccines (Hollingsworth and Jansen, 2019; Mougel et al., 2019). This prompted us to seek a way to induce the activation of immune cells, assist the way of action of immune preparations, and enhance therapeutic effects.

## Natural Polysaccharides in Anti-Tumor Activity

We searched PubMed and Google Scholar using keywords such as “polysaccharide,” “macrophage cell,” “dendritic cell,” “NK cell,” “cancer immunotherapy,” among others. From the retrieved literature, we summarized the regulatory mechanism of plant polysaccharides represented by Chinese herbal and fungal polysaccharides in tumor immunotherapy.

It is reported that more than 300 kinds of bioactive polysaccharides have been isolated from natural products. According to the sources, they can be divided into five categories: fungal, higher plant, lichen and algae, animal, and bacterial polysaccharides (Yin et al., 2019). Polysaccharides are generally composed of glycosidic covalently linked monosaccharides and contained different proportions of mannose, galactose, glucose, xylose, arabinose, rhamnose and other types of monosaccharides (Pang et al., 2018). At present, researches are focused on arabinogalactan, galactomannan, and pectin polysaccharides from higher plants, β-glucan from fungi, and sulfated polysaccharides from seaweed (Yu et al., 2018). Plant polysaccharides contain a variety of skeleton structures and have great potential for structural diversity so that they have the most powerful ability to carry a large amount of biological information (Jiao et al., 2016). In addition, several laboratory studies have shown that fungal polysaccharides have higher anti-tumor functions, while plant polysaccharides may have a better effect of enhancing the immunomodulatory effect (Jiang et al., 2010; Tang et al., 2012; Hou et al., 2020).

**FIGURE 2:**
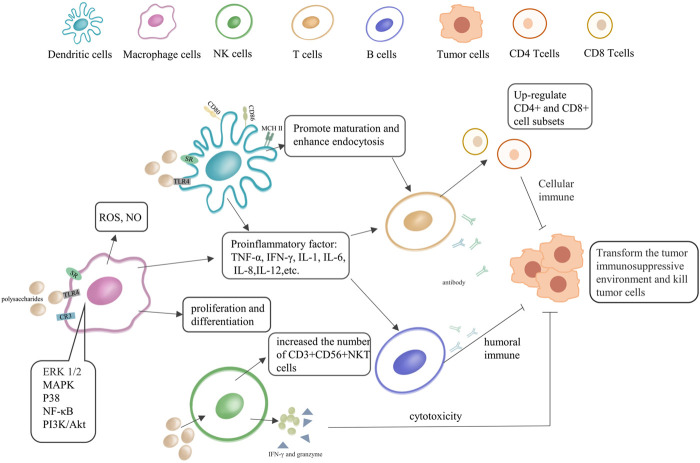
The immunoregulatory effect of natural polysaccharides in immune cells. Polysaccharides can directly or indirectly activate dendritic cells (DCs), NK, macrophages, T and B lymphocytes, immune cells. They can also produce antibodies, secrete immune factors, and promote immune activation pathways.

Previous studies have shown that polysaccharides are involved in the regulation of various immune cell-mediated biological phenomena, having certain anti-tumor and extensive immunomodulatory effects (Jiang et al., 2010). We briefly summarized the regulation process of polysaccharides on innate immunity in Figure 2, and summarized the respective action pathways of plant and fungal polysaccharides in Supplementary Figure S1. Their anti-tumor activity is usually mediated through two main pathways. One refers to the direct suppression/eradication of malignant cells and activation of the innate and/or adaptive immune system, while the other contributes to the activation of various immune cells and increases the production of a series of important immunomodulatory cytokines (Li W. et al., 2019). The binding of polysaccharides to specific receptors [Toll-like receptors 4 (TLR4), scavenger receptor (SR), etc.] on DCs and macrophages promotes their activation and maturation, enhances the production of pro-inflammatory cytokines, stimulating polarization to Th1, induces the activation of antigen-specific CD8+ CTLs, and improves the immunosuppressive state of the tumor microenvironment (Liu et al., 2016). Several researches have shown that polysaccharides stimulate the release of TNF-α and NO in macrophages, and they contribute to the anti-tumor activity and immune regulation of tumor-bearing hosts. Moreover, they can also enhance the antibacterial activity of neutrophils and promote the cytotoxicity of NK cells (Li W. et al., 2019; Bamodu et al., 2019; Del Corno et al., 2020).

In clinical applications, compared with polysaccharides from other sources, plant and fungal polysaccharides have a large amount of medical experience. This review focuses on selecting some representative fungal and plant polysaccharides, discussing their regulatory effects on immune cells, immune molecules, and immune genes at the cellular and molecular levels (Table 1). The potential of polysaccharides as immunomodulators is also discussed.

**TABLE 1:** The immunoregulatory activity of natural polysaccharides and their derivates on immune cells.

	Polysaccharides compounds	Study model	Mechanism and effect	References
Polysaccharides of higher plant origin	*Astragalus* polysaccharides	Non-small cell lung cancer (NSCLC) H441 and H1299 cells	Increase the M1/M2 macrophage polarization ratio; promote the functional maturation of DCs and enhance the T cell-mediated anticancer immune responses	Bamodu et al. (2019)
		4T1 murine and CT26 cells；BABL/c mice	Downregulate the expression of PD-L1 on the cell surface via the protein kinase B (Akt)/mammalian target of rapamycin (mTOR)/ribosomal protein S6 kinase beta-1 (p70S6K) pathway	Chang H.-L. et al. (2020)
		Inbred strain BALB/c mice (approximately 6–8 weeks-old, female); the murine mammary carcinoma 4T1 cells and RAW264.7 cells	Convert macrophages to M1 phenotype, up-regulate the expression of notch ligand and promote the expression of M1 markers of macrophages, including inducible NO synthase, IL-6, TNF-α and CXCL10	Wei et al. (2019)
		BALB/c mice	Enhance the proliferation of spleen lymphocytes and increase phagocytosis of peritoneal macrophages in mice and up-regulate the expression of IL-2, TNF-α and IFN-γ in peripheral blood	Li et al. (2020)
		RAW264.7 and 4T1 cells		
		B6C3F1 mice	Induce nitric oxide (NO) production and inducible NO synthase (iNOS) transcription through the activation of NF-κB/Rel	Lee and Jeon (2005)
		RAW 264.7 cells		
		MCF-7 and RAW264.7 murine macrophage-like cells	Up-regulate the production of NO and TNF-α	Li W. et al. (2019a)
		BALB/c mice	Elevate cytokine and anti-PD-1 antibody titers and response elected	Chang F.-L. et al. (2009)
		C57BL/6j (H-2^b^) mice	Induce maturation of BM-derived DC, increase membrane molecules, including CD11c and I-A/I-E, and IL-12 in DC and reduce the endocytic activity of DC	Shao et al. (2006)
	*Lycium barbarum* polysaccharides	BALB/c mice, murine colon cancer cell line CT26WT	Induce the phenotypic and functional maturation of DCs via notch signaling and promote the cytotoxicity of DC-mediated CTLs	Wang W. et al. (2018)
		RAW264.7 macrophage cells	Activate macrophages by inducing the production of TNF-α and up-regulation of MHC-II costimulatory molecules to enhance innate immune function	Chen et al. (2009b)
		HeLa, HepG2, HEK293 and LoVo cell lines; MCF-7R and A2780T cells; Caco-2 and RAW264.7 cells	Enhance the viability of macrophages RAW264.7 cells and induced cell polarization, regulate the production of NO, TNF-α, IL-6 and ROS in RAW264.7 cells	Feng et al. (2020)
		C57BL/6J (H-2^b^) and BALB/c (H-2^d^) mice	Induce the maturation of dendritic cells and enhance the stimulating activity to allogeneic T cells by up-regulating the expression of CD40, CD80, CD86 and MHCII molecules and down-regulating the antigen uptake of dendritic cells	Zhu J. et al. (2007)
	Angelan	Murine macrophage, RAW264.7 cells	Induce NO production and cytokine gene expression involved in innate immune responses; activate macrophages and DCs to secrete cytokine IL-12 through the TLR4 signaling pathway; induce strong anti-cancer activity of NK and NKT cells *in vivo*	Kim et al. (2018)
		Female C57BL/6 mice; B16F10 murine melanoma cells	Enhance the immune functions of B cells, macrophages, and natural killer cells	Han et al. (2006)
		Female C57BL/6, BALB/c, C3H/HeN and C3H/HeJ mice	Induce DC maturation via TLR4 signaling pathways	Kim et al. (2007)
		Female C57BL/6 mice	Increase the expression of DC maturation markers, through the NF-κB pathway and increase CCR7 expression in DCs; enhance DC homing from tissues to draining lymph nodes *in vivo*	Kim J. Y. et al. (2011)
	*Salvia miltiorrhiza* polysaccharides	Male wistar rats	Stimulate splenocyte proliferation, promoted anti-inflammatory cytokines (IL-2, IL-4 and IL-10) production, inhibited pro-inflammatory cytokine (IL-6 and TNF-α) secretion, augment the killing activity of NK cells and cytotoxic T lymphocytes (CTL), and increase phagocytotic function of macrophages in gastric cancer rats	Wang N. et al. (2014)
		The lymphocytes were obtained from the peripheral blood of cancer patients; cancer cell lines A549, hepG2 and HCT116	Promote the proliferation of T lymphocytes; up-regulate the gene expression of cytokines IL-4, IL-6 and IFN-γ; enhance gene expression of TLR1, TLR2 and TLR4	Chen et al. (2017)
		Mouse hepatocellular carcinoma cells H22	Increase the concentration of TNF-α in serum of H22-bearing mice; improve the spleen index and thymus index and the immune response	Liu et al. (2013)
	*Rehmannia glutinosa* polysaccharides	C57BL/6 and BLAB/c mice	Induce the proliferation of NK cells in mice *in vivo*, promote TLR4-dependent IFN-γ production and CD69 expression and enhance cytotoxic activities and type I IFN production in spleen NK cells	Xu et al. (2017b)
		C57BL/6 (6 weeks old), BALB/c, OT-I and OT-II TCR transgenic mice and C57BL/6-Ly5.1 (CD45.1) congenic mice; TLR2, TLR4 and SR-A-KO mice; the murine melanoma cell line B16F10 (ATCC, CRL-6475) expressing OVA (B16-OVA) and murine carcinoma cell line CT26 (ATCC, CRL-2639)	Increase levels of co-stimulatory molecule expression and pro-inflammatory cytokine production in spleen DCs dependent on TLR4; enhance ovalbumin (OVA) antigen (Ag)-specific immune activation in tumor-bearing mice	Xu et al. (2017a)
		Elutriated PBDCs	Decrease phagocytic activity and increased expression levels of co-stimulatory molecules in MDDCs; elevate the production of proinflammatory cytokines	Wang Y. et al. (2018)
	*Dendrobium* polysaccharides	DEAE52, S200, Ser536, C22B4, Thr180/Tyr182, ERK1/2, Thr202/Tyr204, C-20, F-2, Ser727, C-20, D-2, G-7, PY1007/1,008, PT308; anti-rabbit IgG-HRP (1:5,000), and anti-mouse IgG-HRP (1:5,000) and antigoat IgG-HRP	Evaluate the secretion level of cytokine IL-1β and IL-10 and TNF-α *in vitro*; lead to the phosphorylation of NF-КB and Erk1/2 but also suppress phosphorylation of Erk1/2 in THP-1 cells induced by PMA	He et al. (2016)
		RAW264.7 cell line; NK cells	Stimulate splenocyte proliferation and secrete cytokines IL-2 and IL-4, to activate macrophages to produce NO and cytokines TNF-α and IL-1β; enhance the phagocytosis of RAW267.4 cells significantly and cytotoxicity of natural killer (NK) cell	Xia et al. (2012)
		BALB/c mice (male, 6–8 weeks old, 20 ± 2 g); the CRC mice model was induced by AOM/DSS	Improve the metabolic ability of tumor infiltrated CD8+ cytotoxic T lymphocytes (CTLs) and reduce the expression of PD-1 on CTLs to enhance the anti-tumor immune response in the tumor microenvironments	Liang et al. (2019)
		Human peripheral blood mononuclear cells (PBMC) were isolated from healthy donors; female BALB/c mice	Induce TH1, TH2, inflammatory cytokines and chemokines in mouse *in vivo* and human cells *in vitro*; expand mouse splenocytes *in vivo* including CD4+ T cells, CD8+ T cells, B cells, NK cells, NKT cells, monocytes/macrophages, granulocytes and regulatory T cells	Lin et al. (2014)

	*Ginseng* polysaccharides	YAC-1 cell line; 6-week-old female BALB/c mice	Increase the anticomplementary activity and cytokine production including IL-6, IL-12, and TNF-α; enhance the production of interferon (IFN)-γ and granzyme B of NK cells	Lee et al. (2019a, 2019b)
		HCT-1 16 and HT-29 human colon cancer cells	Inhibit IL-8 secretion and cancer cell proliferation, inhibit CD4^+^IFN-γ^+^cell (Th1) differentiation, and decrease CD4^+^FoxP3^+^cell (treg) differentiation	Wang C.-Z. et al. (2020)
		Male or female C57BL/6 mice (6–8 weeks old, 18–22 g); human erythroleukemia K562 cells; HL-60 cells; KG1α cells	Stimulate macrophage, increase the expressions of CD_68_, ACP and α-ANE in mouse; enhances the levels of cytokines, including TNF-α, IL-1, IL-6 and NO	Wang et al. (2010)
		C57BL/6 mice; bone marrow cells were harvested from femur and tibia of C57BL/6 mice	Enhance the expression of CD86 on DC surfaces and stimulate proliferation of allogeneic CD4^＋^ T lymphocytes	Kim et al. (2009)
Polysaccharides of fungal origin	*Ganoderma lucidum* polysaccharides	BALB/c mice	Stimulate B cell proliferation and activation, promotes T cell release of TNF-α and IFN-γ, enhance activation and maturation of immature DC, promote macrophage differentiation and maturation, and sensitized NK cell-mediated cytotoxicity	Xu et al. (2011)
		RAW264.7 cells		
		Lewis lung cancer model	Increase the percentage of CD4+ and CD8+ T cells together with the production of Th1-type cytokines (IFN-γ and IL-12) in the spleen	Wang Y. et al. (2020)
		BALB/C mice RAW264.7 cells	Induce enhancement of phagocytosis and increase in NO release and cytokines IL-6 and TNF-α production, increase the phosphorylation level of phosphorylated MAPKs	Li et al. (2018)
		4T1 cells		
		C57BL/6J mice	Increase the expression of both TNF-α and IFN-γ (at both mRNA and protein levels) in splenocytes and increase cytotoxic T lymphocyte cytotoxicity and NK activity	Gao et al. (2005a)
		Sarcoma180 cells		
		C57BL/6 (H-2^b^) mice	Suppress the production of IL2, IFN-γ and TNF-α in mononuclear lymphocytes by B16F10 cell culture supernatant; restore the phagocytosis activity of macrophages and the production of NO, TNF-α; enhances MHC class I molecules and costimulatory molecules	Sun et al. (2012), Lu et al. (2014), Sun L.-X. et al. (2015)
		B16F10 melanoma cells		
		The mouse hepatoma H22 cell line and normal hepatic cell line L-02; kunming and BALB/c male mice	Increase the ratio of Teffs (effector T cell) to Tregs; inhibit the expression of Notch1 and FoxP3 through increase of miR-125b expression	Li et al. (2015)
		Patients with lung cancer	Stimulate the activation of lymphocytes, increases the production of CD69, perforin and granzyme B	Gao et al. (2005b), Sun et al. (2014)
		Inbred strain male (6–8 weeks old) C57BL/6j (H-2^b^) mice	Promote NK cells and NKT cells in the spleen of mice, enhance the activity of cytotoxic T lymphocytes, and promote the phagocytosis and cytotoxicity of macrophages	Zhu X.-L.et al. (2007)
	*Ganoderma atrum* polysaccharides	CT26 WT mouse colon cell line; BALB/c mice	Activate macrophages through TLR4-dependent signaling pathways; induce apoptosis	Zhang et al. (2013, 2014b)
		The Murine Sarcoma cell line (S180)	Enhance the induction of apoptosis through cAMP-PKA signaling pathway and down-regulation of Ca2+/PKC signal pathway; promote lymphocyte proliferation and macrophage phagoctytic activity	Zhang et al. (2014a)
	*Ganoderma sinense* polysaccharides	RAW264.7 macrophages	Upregulate TLR4 protein expression and activates the MAPK pathway; induce the production of the cytokines TNFα, IL-1β, and IL6	Liu et al. (2019)
		Human peripheral blood mononuclear cells	Enhance the productions of TNF-α, IL-10, and TGF-β; increase the IL-10 and IL-12 productions in MDDC	23859044 (Yue et al. 2013)
	*Ganoderma formosanum* polysaccharides	Male C57BL/6 and BALB/c mice, CB17-SCID mice; murine sarcoma 180 and B16 melanoma cells, C26 adenocarcinoma cells	Promote the maturation of DC and Th1-polarized adaptive immune response; stimulate both the production of natural antitumor antibodies and the activation of CR3+ leukocytes	Wang C.-L. et al. (2014)
		Male C57BL/6 mice, OT-II TCR transgenic mice and C57BL/6-Ly5.1 (CD45.1) congenic mice; MO5, an OVA gene-transfected B16 melanoma cell line and EL4 cells	Induce a Th1-polarized adaptive immune response; stimulate dendritic cells to mature and produce pro-inflammatory cytokines	Pi et al. (2014)
	Lentinan	Tumor-bearing mice (P-8l5-DBA/2)	Increase the generation of cytotoxic T lymphocytes (CTL)	Chihara (1983)
		Colon-26, meth A, YAC-1	Increase the frequency of tumor-infiltrating CD86+ cells, augment T-cell stimulating activity of the host’s own DCs, and induce tumor-specific CTLs; improve the modification of the Th1/Th2	Mushiake et al. (2004)
		NSCLC patients treated with NP chemotherapeutic protocol	Increase CD3+ CD56+ NKT cells; inhibit the expansion of immune suppressive tregs; down-regulate the percentage of CD4+ CD25+ tregs, leading to a shift in the inflammatory status from Th2 to Th1	Wang X. et al. (2018)
	*Poria cocos* polysaccharides	The murine macrophage cell line RAW 264.7	Increase the production of nitric oxide (NO), TNF-α, IL-1β, IL-6 and intracellular calcium level	Pu et al. (2019)
		Female C57BL/10ScNJ and control C57BL/10J; RAW 264.7 cells; Lewis lung cells	Increase levels of nitric oxide, IL-2, IL-6, IL-17A, TNF, and IFN-γ	Tian et al. (2019)
		C57BL/6J mice between 6 and 8 weeks old as wild-type (WT) controls; TLR2-deficient and TLR4-deficient mice; the murine macrophage cell line RAW 264.7	Stimulate RAW 264.7 macrophages *in vitro* through the induction of TNF-α, IL-1β and the regulation of NF-κB-related gene expression; activate peritoneal cavity macrophages to induce TLR4-mediated myeloid differentiation factor 88 (MyD88)-dependent signaling	Chang et al. (2009)
Polysaccharides derivatives and composite materials	The combination of gold nanoparticles with *astragalus* polysaccharides (APS-AuNP)	4T1-bearing mice model	Induce dendritic cells maturation through phenotypic markers with functional changes, promote T-cell proliferation and enhance cytotoxicity; increase the population of CD4/CD8 T lymphocytes	Pang et al. (2019)
	The sulfated polysaccharides (SPS) from marine macro algae	RAW 264.7 cells	Stimulate macrophage proliferation and production of prostaglandin and nitric oxide, COX-2, 5-LOX, and iNOS; enhance the mRNA expression of pro- inflammatory cytokines and anti-inflammatory cytokines	Jose et al. (2017)
	Sulfated polysaccharides of lentinan	14-day-old chickens	Enhance serum antibody titer and promote lymphocyte proliferation	Guo et al. (2009)
	Gold nanocomposites containing *Ganoderma lucidum* polysaccharides	4T1 tumor-bearing mice	Induce dendritic cell (DC) activation and promote the proliferation of CD4+ and CD8+ T cells via DC	Zhang et al. (2019)
	Wrapping the *Angelica sinensis* polysaccharides and model protein antigen ovalbumin into poly (lactic-co-glycolic acid)	BALB/c mice; splenic lymphocytes were isolated from the immunized mice on days 21 and 28	Enhance lymphocyte proliferation and improve the ratio of CD4 to CD8 T cells; induce vigorous and long-term IgG immune responses with a mixed Th1 and Th2 responses and up-regulate the levels of Th-associated cytokines	Gu et al. (2019)

### Plant Polysaccharides

#### *Astragalus* Polysaccharides

*Astragali Radix* (Huangqi in Chinese), the dried root of *Astragalus membranaceus,* a commonly used Chinese medicine, has been proven to be effective in enhancing the immune system and treating pathological diseases, even cancer (Chang F.-L. et al., 2020). The *astragalus* polysaccharide (APS) is the main active component of *A. membranaceus* extract, and the combined treatment strategy used with other drugs (such as cancer chemotherapy and immunosuppressants drugs) has also been proved to obviously reduce the toxicity of these drugs and enhance the therapeutic effect (Li et al., 2020).

Bamodu et al. (2019) proved that *astragalus* polysaccharides can significantly increase the polarization rate of M1/M2 macrophages in non-small cell lung cancer (NSCLC) cell lines, regulate the M1/M2 macrophage pool (M1 macrophages produce pro-inflammatory factors and enhance the expression of MHC-II and costimulatory molecules), enhancing the body’s immune response. Using the results *in vitro* of the clinical samples of the NSCLC cohort it was confirmed that APS can also promote the functional maturation of DCs, enhance the T cell-mediated anti-cancer immune response, and synergistically enhance the therapeutic effect of cisplatin, which proves that it is a substitute in the clinical feasibility of immunotherapy. In addition, further studies by Wei et al. (2019) showed that APS induced increased gene expression of M1 markers (including iNOS, IL-6, TNF-α and CXCL10), and induced macrophages to polarize to the M1 phenotype through the notch signaling pathway. In addition, APS can activate macrophages and release NO and TNF-α by activating TLR4 and NF-κB/Rel, directly preventing the growth of cancer cells (Li W. et al., 2019). Furthermore, research by Lee and Jeon (2005) showed that APS stimulate macrophages to express the iNOS gene by activating NF-κB/Rel.

Shao et al. (2006) confirmed through mice experiments that APS-treated DCs secreted higher levels of IL-12, and showed a more mature state, with long protuberances, while untreated DCs showed shorter protuberances than stimulated DCs. Besides, it was confirmed that APS could up-regulate the membrane expression of MHC and costimulatory molecules on DC. Du et al. (2012) have also proved this point, and believed that APS can further induce CD4+T cells to produce IL-4, IL-2, and IFN-γ, enhance the expression of IFN-γ in CD8+T cells, and induce the strong activity of CTL. These studies also make APS a potential herbal medicine that can be used to increase the anti-tumor effect of DC therapy.

In recent years, with the rise of immune agents, the immunomodulatory function of traditional Chinese medicine on immune checkpoints has also aroused great interest. Previous studies (Chang F.-L. et al., 2020) have shown that APS can significantly inhibit the growth of melanoma cells in transgenic mice and reduce the expression of PD-L1 in tumors, suggesting that the anti-tumor immunosuppressive mechanism of APS may also be related to the regulation of PD-1/PD-L1 signal pathway. A study demonstrated that there was no significant difference in tumor inhibition compared with anti-PD-1 combined with ixabepilone or APS, indicating that APS can maintain an effective dose of anti-PD-1 antibody *in vivo*, and delay the progression of tumor or tumorigenesis by increasing the activity of T cells, which may improve the synergistic effect (Chang F.-L. et al., 2020). In addition, another research showed that APS enhances the chemotherapy by stimulating host immunity through reducing the PD-L1 expression of tumor surface (Chang H.-L. et al., 2020). It can reduce the expression of pro-inflammatory cytokines, promote the maturation of DCs and it also reduced the M2 macrophage population in patients with lung cancer (Huang et al., 2019). Based on the above data, we have reasons to think that APS can be used in conjunction with ICI to improve the immune cell infiltration and enhance therapeutic effects.

#### *Lycium barbarum* Polysaccharides

The *Lycium barbarum* polysaccharides (LBP) are one of the main active components in the *Lycium barbarum* fruit (Goji in Chinese)*.* It is a mixture of proteoglycans and polysaccharides and their biological activities are mainly reflected in the antioxidant, anticancer, regulation of immune activity and cytoprotective effects on normal cells (Ho et al., 2009; Wu et al., 2010). Several studies have confirmed that administration of LBP can induce phenotypic and functional maturation of DCs, induce immunogenicity, enhance Th1 response and activate T lymphocytes (Chen et al., 2009a; Wang W. et al., 2018).

Chen et al. (2009b) have shown that LBP can activate macrophages by activating transcription factors NF-κB and AP-1, can induce the production of TNF-α, and up-regulate MHC II molecules. Feng et al. (2020) also observed that LBP significantly enhanced the release of NO, TNF-α, and IL-6 from RAW264.7 macrophages, and significantly promoted the production of ROS. During phagocytosis, it can help eliminate intracellular pathogens (Khatua and Acharya, 2019). Furthermore, LBP induce the maturation of dendritic cells and enhance the stimulating activity to allogeneic T cells (Zhu J. et al., 2007; Tang et al., 2012). In addition, LBP increased the expression of IL-2 and the TNF-α in both mRNA and protein levels of human peripheral blood mononuclear cells (Gan et al., 2003). It is suggested that the combination of LBP and subunit vaccine with weak immunogenicity can improve the level of protective immune response. Furthermore, polysaccharides can not only improve the anti-tumor capabilities, but also avoid tissue damage caused by superfluous production of inflammatory factors (Graff et al., 2009; Liu et al., 2018).

These results suggest that an appropriate amount of LBP not only has the potential to be used as an immunologic adjuvant, but also can prevent immune injury caused by excessive activation of macrophages (Feng et al., 2020). In addition, LBP can be used as a very valuable adjuvant to cancer therapy (such as chemotherapy, radiotherapy, and immunotherapy), reducing the side effects of other types of cancer mediated by apoptosis, and enhancing the antineoplastic effects of other forms of cancer (Tang et al., 2012).

#### Angelan

Angelan is a type of polysaccharide isolated from the water-soluble part of the *Angelica sinensis* (Oliv.) Diels (Dang Gui in Chinese) extract. It can activate both the innate and the acquired immune system, enhance the immune function of B cells, macrophages, and natural killer cells, indirectly activate cytotoxic and helper T cells, and directly inhibit cancer cell adhesion to inhibit tumor growth and metastasis (Han et al., 2006; Kim et al., 2018).

Han et al. (2006) found that Angelan can significantly prolong the survival rate and reduce the frequency of lung metastasis in melanoma transplanted mice, and combined with doxorubicin, it can significantly enhance the therapeutic effect and inhibit tumor growth. Moreover, Kim et al. (2007) found that a mature DC treated with Angelan was more effective than an immature DC in inhibiting the growth of B16F10 tumor in the syngeneic murine tumor model. The results showed that Angelan induces DC maturation through the TLR4 signal pathway, suggesting that Angelan may be used in DC immunotherapy. After further study, they found that *Angelica* polysaccharides up-regulate the expression of MHC-I/II, CD80 and CD86 through the NF-κB pathway, increase the expression of CCR7 in DCs, promote the maturation of DC and the migration of CCL19 *in vivo*. In the B16-F10 syngeneic tumor model, Angelan can enhance the anti-tumor activity of DC (Kim J. Y. et al., 2011). These data show that Angelan can help overcome the shortcomings of DC-based cancer immunotherapy and enhance the therapeutic effect.

#### *Salvia miltiorrhiza* Polysaccharides

The *Salvia miltiorrhiza* polysaccharide (SMP) is a kind of natural polymer that has antioxidant properties and immunomodulatory activity (Wang N. et al., 2014). SMP has been proved to stimulate the proliferation of T lymphocytes in mice, activate the host’s collective immune response, and play an anti-tumor effect (Chen et al., 2017). Furthermore, there are no obvious side effects. A study (Liu et al., 2013) showed that SMP can significantly increase spleen and thymus index of mice, and improve the ability of immune regulation. Chen et al. (2017) used T lymphocytes and homologous tumor cell lines from patients with lung, liver, and colon cancer to demonstrate that SMP specifically promotes the proliferation of peripheral blood T lymphocytes and enhances their cytotoxicity in tumor patients in a dose-dependent manner. In addition, the gastric cancer rats experiments (Wang N. et al., 2014) showed that SMP significantly stimulates the proliferation of splenocytes, promotes the production of anti-inflammatory cytokines (IL-2, IL-4 and IL-10), inhibits the secretion of pro-inflammatory cytokines (IL-6 and TNF-α), enhances the killing activity of NK cells and cytotoxic T lymphocyte (CTL), as well as promotes the phagocytosis of macrophages in them. Furthermore, SMP significantly increased the total intracellular granzyme B and IFN-γ. These studies suggest that the addition of SMP to the treatment can improve the immune function and help improve the quality of life of patients. Therefore, SMP is also a valuable immunomodulator for the treatment of tumors with immunosuppression.

#### *Rehmannia glutinosa* Polysaccharides

The *Rehmannia glutinosa* polysaccharide (RGP), one of the active components of *R. glutinosa* Libosch*.*, has strong immunomodulatory properties and anti-tumor activities, and it is involved in maintaining homeostasis *in vivo* (Zhang et al., 2008). RGP stimulate host immune response by inducing dendritic cell maturation and activating NK cells to induce anticancer effect.

Xu et al. (2017b) have verified the stimulating effect of RGP on NK cells and DCs. RGP can induce the number of NK cells in the peripheral blood and the proliferation of NK cells in the spleen and whole blood of C57BL/6 mice. Furthermore, the RGP treatment also promoted the production of IFN-γ dependent on TLR 4 and the up-regulation of CD69 expression on NK cells in splenic. The killing activity of NK cells treated with RGP against Yac-1 cells was enhanced, and the production of I-IFN finally inhibited the growth of CT26 lung tumor. In addition, tumor-bearing mice experiments showed that RGP could induce the expression of DC costimulatory molecules and the production of pro-inflammatory cytokines in splenic dendritic cells dependent on TLR4, enhance the antigen presentation of DC, and promote the production of IFN-γ by CD4 and CD8T cells. Moreover, the combination of RGP and Ag effectively inhibited the growth of CT26 tumor and B16 melanoma in BLAB/c and C57BL/6 mice. RGP induces the maturation of dendritic cells and activates antigen specific immune response in tumor-bearing mice, which promotes the infiltration of T cells into tumor (Xu et al., 2017a). Subsequently, Wang Y. et al. (2018) confirmed this in human cell experiments *in vitro*. RGP treatment significantly decreased the phagocytic activity of MDDC and induced the activation of T cells. Besides, RGP can upregulate costimulatory molecules and production of proinflammatory cytokines in both MDDC and PBDC subsets. These data suggest that RGP may play a role as an immunostimulatory molecule and it is expected to be used as an effective adjuvant in the immunotherapy of tumors.

#### *Dendrobium* Polysaccharides

*Dendrobium* polysaccharide, one of the main active components of the traditional Chinese herbal medicine “*Dendrobium*,” has the effects of antioxidation, immune enhancement, and anti-tumor. Researchers compared different kinds of *Dendrobium* from different producing areas and found that most of the crude polysaccharides or purified polysaccharides extracted from *Dendrobium* plants can enhance the function of immune cells, increase the secretion of cytokines, activate macrophages. In particular, the polysaccharides extracted from *Dendrobium officinale* polysaccharide (DOP) and *Dendrobium huoshanense* polysaccharide (DHP) have better immunomodulatory activity (Meng et al., 2013; Yue et al., 2020).

Several studies have shown that DOP can stimulate the proliferation of splenocytes, and secrete cytokines IL-2 and IL-4, to induce morphological changes of macrophages, thus promoting the production of cytokines TNF-α, IL-6, IL-1 β, and NO. Further, they enhance the phagocytic activity of RAW267.4 macrophages and significantly enhance the killing activity of NK cells, which may be involved in the early immune response (Xia et al., 2012; He et al., 2016; Yue et al., 2020). In addition, it was observed that DOP could inhibit the growth of tumor cells in mice with sarcoma 180 cells, which can be related to its mechanism (Yue et al., 2020). Liang et al. (2019) found in the colorectal cancer model that DOP can enhance the metabolic ability of tumor infiltrating CD8^+^ CTL, reduce the loss of mitochondria the expression of PD-1 on CTL, thus enhancing the anti-tumor immune response of TME.

DHP can induce the production of Th1, Th2, inflammatory cytokines, and chemokines in mouse and human cells *in vivo* and *in vitro*. Additionally, it expanded mouse spleen cells *in vivo*, including CD4+T cells, CD8+T cells, B cells, NK cells, monocytes/macrophages, granulocytes and Tregs, and had strong anti-inflammatory ability (Lin et al., 2014). These data show that DHP can be used as an immune synergistic agent to exert its potential in immunotherapy in the future.

#### *Ginseng* Polysaccharides

According to the different extraction parts, we divided the ginseng polysaccharides into polysaccharides extracted from the roots of *Panax ginseng* (GSP) and polysaccharides extracted from the fruits of *Panax ginseng* (GBP). Some studies have shown that the berry of *Panax ginseng* has a much stronger pharmacological activity than its root (Lee et al., 2019a; Lee et al., 2019b).

The anticancer effect of GBP isolated from ginseng berries may be due to the increased activity of innate immune cells, such as macrophages and NK cells (Lee et al., 2019a). GBP can promote the production of IL-6, IL-12, and TNF-α as well as increase the expression of mRNA in mouse peritoneal macrophages. Furthermore, it significantly increases the killing activity of YAC-1 tumor cells to NK cells and the production of granzyme B. It can also inhibit the lung metastatic activity of B16-BL6 melanoma cells (Lee et al., 2019a; Lee et al., 2019b). In addition, GBP can significantly inhibit the differentiation of Th1 cells and the differentiation of Treg cells (hindering the body’s immune response to malignant tumors), and the differentiation of Treg cells Down-regulation may help the anti-cancer potential of GBP and reshape the tumor microenvironment (Wang C.-Z. et al., 2020).

However, ginseng polysaccharides (GSP) mostly regulate immunity through macrophages and dendritic cells. Wang et al. (2010) showed that mouse peritoneal macrophages (PMs) treated with GSP could induce tumor killing activity, enhance phagocytic activity and the expression of CD68 and show the ability to produce cytokines and cytotoxic molecules. Kim et al. (2009) proved that GSP can induce the maturation of mouse bone marrow-derived DC, enhance the expression of CD86 on the surface of DCs, and significantly promote the proliferation of allogeneic CD4+T lymphocytes.

Taken together, these results suggest that ginseng polysaccharides have strong anti-tumor activities by stimulating macrophages and they may act as an immunomodulator against diseases such as cancer.

### Fungal Polysaccharides

#### *Ganoderma* Polysaccharides

The main active components of *Ganoderma* are polysaccharides and triterpenoids, among which the polysaccharide fraction is responsible for antitumor and immunomodulatory effects. The main mechanism of anti-tumor activity of *Ganoderma* polysaccharides is stimulating the host’s defense response, activating lymphocytes to enhance the immunogenicity of tumor cells, rather than killing tumor cells directly. However, *Ganoderma* plants are rich in species and widely studied. Therefore, we will divide it into the following 4 parts to elaborate: *Ganoderma lucidum* polysaccharide (GLP), *Ganoderma atrum* polysaccharide (PSG-1), *Ganoderma sinense* polysaccharide (GSP-2) and *Ganoderma formosanum* polysaccharides (PS-F2).

GLP, a polysaccharide extracted from *Ganoderma lucidum*, can regulate the function of a variety of immune cells, including macrophages, dendritic cells, NK cells, T cells, and B cells. It can stimulate the proliferation and activation of B cells, promote the release of TNF-γ and IFN-α from T cells, enhance the activation and maturation of immature DCs, increase the phosphorylation level of MAPK and promote the differentiation and maturation of macrophages, and sensitize NK cell-mediated cytotoxicity (Xu et al., 2011). GLPs can effectively promote the activation and maturation of immature DCs, and they are more inclined to Th1 reaction (Zeng et al., 2019).

We further summarized the regulatory role of GLP in tumor microenvironment in different cancers. Li et al. (2018) found that in 4T1 breast cancer BALB/c mice, GLP can significantly inhibit tumor growth and induce macrophages to inhibit the survival and migration of cancer cells *in vitro*. For lung cancer, GLP antagonized the immunosuppression of lung cancer tumor cells and stimulated the activation of lymphocytes in lung cancer patients to improve immune function (Gao et al., 2005b; Sun et al., 2014). A research on lewis lung cancer mouse (Wang Y. et al., 2020) further concluded that GLP can inhibit tumor growth and regulate the differentiation and inhibition of myeloid-derived suppressor cells (MDSCs) to enhance antitumor immune response. After GLP treatment, there were fewer MDSCs in both spleen and tumor tissues and the production of Th1-type cytokines together with the percentage of CD4+T and CD8+T cells was increased in the spleen of mice, indicating a better immune infiltration microenvironment. For melanoma, several studies (Sun et al., 2012; Lu et al., 2014; Sun L.-X. et al., 2015) have shown that GLP can completely or partially improve the inhibitory effect of B16F10 cells on the production of IL-2, IFN-γ and TNF-α by mononuclear lymphocytes in the culture supernatant of B16F10 cells. In addition, it can also promote the proliferation and activation of lymphocytes induced by melanoma cells, increasing the production of CD69 and IFN-γ, and enhancing the expression of MHC-I and costimulatory molecules to induce more effective immune cells mediated cytotoxicity and control tumor progression. Then, GLP can inhibit the accumulation of Treg and inhibit the growth of liver cancer by inducing miR-125 in hepatoma-bearing mice (Li et al., 2015).

In addition, GLP can also antagonize the immunosuppressive effect caused by drugs by restoring the function of immune effector cells (including macrophages, NK cells and NKT cells) (Zhu X.-L. et al., 2007). Therefore, when combined with chemotherapy or other therapies, GLPs can alleviate treatment-induced immunosuppression, enhance the anti-cancer effect of them and improve the health status (Zhu X.-L. et al., 2007; Zeng et al., 2019).

PSG-1, a polysaccharide extracted from *Ganoderma atrum*, may against drug-induced immunosuppression by increasing levels of TNF-α and IL-2 and promoting immune effort cell survival (Li et al., 2017). In CT26-bearing mice, PSG-1 induced apoptosis by enhancing the antitumor immune response and activate macrophages through TLR4-dependent signaling pathways to inhibit tumor growth (Zhang et al., 2013; Zhang et al., 2014b). In another studies, PSG-1 can increase cAMP and PKA activities and promote lymphocyte proliferation and macrophage phagocytic activity to activating host immune function in tumor-bearing mice (Zhang et al., 2014a).

GSP-2, a polysaccharide extracted from *Ganoderma sinense*, which specific induced the overexpression of TLR4 and activated the MAPK pathway, promotes cytokine secretion and immune modulation in macrophages (Liu et al., 2019). It can stimulate the proliferation of PBMC and increase the secretion of TNF-α, IL-10 and transforming growth factor-β and enhance the ability of monocyte-derived DCs to produce IL10 and IL-12(Yue et al., 2013). These findings suggested that GSP-2 could be used as an adjuvant in immunosuppressed tumor patients.

PS-F2, a polysaccharide extracted from *Ganoderma formosanum*, stimulates tumor-specific cellular and humoral immune responses by promoting the maturation of DC and Th1-polarized adaptive immune response (Wang C.-L. et al., 2014). At the same time, the adjuvant function of PS-F2 was also investigated in another experiment, which demonstrated that PS-F2 stimulated dendritic cells to mature and produce pro-inflammatory cytokines *in vitro*. Their studies also confirmed that PS-F2, when used as an adjuvant, can play a role in anti-tumor vaccines by inducing a Th-1polarized adaptive immune response (Pi et al., 2014).

In short, polysaccharides extracted from *Ganoderma* have almost no adverse effects on the human body, and other immune enhancers rarely have this advantage. They can regulate the activities of neutrophils, NK cells, NKT cells, dendritic cells, and the complement system, enhancing the immune response and anti-tumor activity (Gao et al., 2005a). In addition, the ability to reverse the immunosuppressive microenvironment caused by multiple drugs also makes it a potential choice of adjuvant for tumor immunotherapy.

#### Lentinan

Lentinan (LNT) is a compound extracted from the edible mushroom *Lentinus edodes*, which has a direct anti-tumor effect and a type of immunomodulatory activity. Its active component is β-(1→3)-D-glucan. It can activate nonspecific cytotoxicity *in vivo* and enhance cytotoxic T cell activity, NK cell activity, and humoral immune response mediated by helper T cells, induce Th1 polarization, and improve the balance between Th1 and Th2 (Chihara, 1983; Ina et al., 2013). It is reported that LNT has been used as a biological response regulator for cancer chemotherapy, being able of improving the quality of life and prolong the survival time of patients (Mushiake et al., 2004).

It has been previously proved that CSF increases after the application of lentinan *in vivo*, which may act on immunomodulatory macrophages, resulting in an increase in the production of IL-1, thus activating the function of helper T cells. In the tumor-bearing environment, most of the immune cells are in a state of inhibition, while lentinan can restore the immunosuppression of allogeneic reactive killer cells to the normal level, and the killer cells produced by spleen cells can also recover from zero to about 40%. Lentinan is the first adjuvant proved to enhance cytotoxic T lymphocyte response *in vivo* (Chihara, 1983). In an animal experiment (Mushiake et al., 2004), lentinan was proved to increase the number of CD86 ^+^ cells infiltrated by tumor to activate DC function. In addition, lentinan can stimulate the production of killer T cells and NK cells, restore the ratio of killer/suppressor T cells, and up-regulate the killing effect on tumor cells mediated by NK cells (Ina et al., 2013).

The combination of lentinan with leukocytes can enhance the cytotoxicity mediated by ADCC and complement by activating CR3, induce the production of IL-12 and enhance the anti-tumor effect of mAbs. A study *in vivo* has clearly shown that lentinan combined with trastuzumab can significantly inhibit tumor growth (Cheung et al., 2002). Many studies have shown that lentinan, as a non-specific BRM, has an effect on the immune regulation of various cancers (including gastrointestinal cancer, breast cancer, lung cancer): the survival rate and quality of life have been significantly improved during the one-year follow-up, and the short-term evaluation of the objective response and disease progression has also been significantly improved. Moreover, lentinan was associated with a lower incidence of adverse events than chemotherapy alone (Ina et al., 2013; Wang et al., 2017; Zhang et al., 2018). *In vitro*, the antitumor effect of LNT was also significantly enhanced when combined with monoclonal antibody and gemcitabine (Hong et al., 2004; Sun M. et al., 2015).

Furthermore, Wang X. et al. (2018) showed that in patients with NSCLC, lentinan significantly increased the number of CD3+CD56+NKT cells, up-regulated CD4+ and CD8+ cell subsets, increased the levels of IFN-γ, TNF-α, and IL-12, and decreased the levels of IL-10 and TGF-β1 in patients with NSCLC. It is confirmed that lentinan can not only enhance the cellular immune function and promote anti-tumor benefit through combined immunotherapy, but also inhibit the expansion of immunosuppressive Tregs. Lentinan-based chemical immunotherapy is a promising anti-tumor strategy by promoting the proliferation of cytotoxic T cells and then increasing the inflammatory chemokines/cytokines. At the same time, the proportion of CD4+CD25+Tregs in NSCLC patients treated with lentinan was down-regulated, resulting in the transformation of the inflammatory state from Th2 to Th1. In view of these, a synergistic effect of immunotherapy with lentinan and monoclonal antibody can be expected.

#### *Poria cocos* Polysaccharides

The *Poria cocos* polysaccharides (PCPs), the main bioactive components extracted from the sclerotia of *Poria cocos* (Schw.) Wolf, are composed of ribose, arabinose, xylose, mannose, glucose, and galactose. Studies have shown that PCPs have anti-tumor, immunomodulatory, antioxidant, and mitogenic effects (Li X. et al., 2019; Pu et al., 2019).

PCP can enhance the innate immunity, improve the proportion of lymphocytes, enhance the phagocytosis of macrophages by activating varieties of immune cells, and regulate the specific immunity by activating T cells. Tian et al. (2019) found that PCPs can directly interact with the surface TLR of macrophages, induce the secretion of NO, IL-2, IL-6, TNF, IFN-γ, and IL-17A, increase the organ immune activity index, play an immunomodulatory role, and reduce the tumor burden. In different animal models, PCPs combined with chemotherapeutic drugs, such as 5-FU (Li X. et al., 2019), can further improve the therapeutic effects and reduce the adverse reactions associated with chemotherapeutic drugs. Chang et al. (2009) found that PCPs can stimulate RAW264.7 macrophages *in vitro* by inducing TNF-α and IL-1β as well as by regulating the expression of NF-κB-related genes. Therefore, PCP can be considered as a new and potential immune stimulant.

### Polysaccharide Derivatives

Polysaccharide has almost no cytotoxic effect on human body, and its safety is extremely high, but it also has significant defects: rapid elimination, short half-life and lack of targeting *in vivo*, which may hinder its sustained pharmacological activity and prevent it from exerting its due effect (Pang et al., 2019). Many researches have shown that polysaccharides with functional groups have more immunostimulatory activity than those without functional groups (Ferreira et al., 2015). The structural modification of polysaccharides can significantly improve immune activity. In the aspect of chemical modifications, we screened sulfation, carboxymethylation, acetylation and phosphorylation as representatives to review the effects (Chen and Huang, 2018).

Sulfated polysaccharides can enhance the phagocytic function of macrophages, stimulate macrophages to secrete NO, IL-6, and other interleukin, and enhance the ability of immune regulation (Kim J.-K. et al., 2011; Jose et al., 2017). The sulfation treatment could significantly improve the immune enhancement activity of seaweed polysaccharides (Jose et al., 2017) and lentinan (Guo et al., 2009). In addition, the sulfated function groups of polysaccharides can enhance cytotoxicity, which are related to the substitution position of its groups: substitution on C-6 > C-4> C-2 of galactose > C-2 of anhydrogalactose (Liang et al., 2014). Furthermore, the Carboxylation and acetylation groups can enhance the water solubility of polysaccharides (Xu et al., 2019). When the degree of substitution of carboxymethyl is 0.5–0.6, carboxymethylation can significantly improve the maturation induction ability of DCs and gain an immunomodulatory effect (Huang G. et al., 2016). The modification of the phosphate group will significantly enhance the expression of B cells and DCs on the surface of CD86 and CD69, and promote the production of IL-10, enhancing the immunosuppressive activity of polysaccharides (Chen and Huang, 2018).

### Natural Polysaccharides Composite Materials

Beyond the modification of derivatives, the rise and application of new materials also provide a wide range of research directions for the modification of natural polysaccharides. The addition of nanocomposites may enhance their pharmacological activities (Pang et al., 2019). The nano-drug delivery system has been widely used in the targeted drug delivery system, and it shows advantages in tumor therapy (Chen and Huang, 2018; Yu et al., 2018).

Compared with other nanoparticles (NPs), gold nanoparticles (AuNPs) have a high surface area-to-volume ratio, dispersion, stability, and biocompatibility, therefore, it is one of the most promising ways for the application of nanotechnology (Cabuzu et al., 2015; Okoampah et al., 2020). The combination of gold nanoparticles with *astragalus* polysaccharides (APS-AuNP) can give *astragalus* polysaccharides longer peripheral circulation and more aggregation in immune organs or tumors, resulting in stronger regional and systemic anti-tumor effects. It can not only induce phenotypic maturation and functional changes of DCs, but also further promote T cell proliferation and cytotoxicity. Compared with free APS and other AuNP, APS-AuNP has a stronger immunomodulatory effect on DCs. Further, animal experiments have confirmed that APS-AuNP has excellent efficacy in inhibiting primary tumor growth and reducing lung metastatic nodules, and it has a significant ability to assist tumor microenvironment reconstruction and systematic anti-tumor immune response (Pang et al., 2019). Besides APS-AuNP, Zhang et al. (2019) have also shown that gold nanocomposites containing *Ganoderma lucidum* polysaccharides (GLP-Au) can effectively induce the activation of DCs, increase the expression of CD80/CD86/CD40/MHCII, and promote the proliferation of CD4+ and CD8+T cells in splenocytes. The combination of GLP-Au and doxorubicin could strongly inhibit the tumor growth and lung metastasis of 4T1, restore the weight loss caused by doxorubicin, and increase the percentage of memory T cells in CD4+/CD44+.

Moreover, there is a vaccine delivery system based on NPs, which can control the release of antigens and promote the immune response to cancer. A novel NPs-based vaccine delivery system (ASP-PLGA/OVA) can be prepared by wrapping the immunopotentiator *Angelica sinensis* polysaccharide (ASP) and model protein antigen ovalbumin (OVA) into poly (lactic-co-glycolic acid; PLGA). Mice treated with ASP-PLGA/OVA nanoparticles can promote lymphocyte proliferation and increase the ratio of CD4/CD8T cells, thus inducing a strong cellular immune response. ASP-PLGA/OVA nanoparticles can induce a strong and persistent immune response to Th1/Th2 mixed response and up-regulate the level of Th-related cytokines (Gu et al., 2019).

The above studies show that the new material of polysaccharides can make up for the shortcomings of natural polysaccharides to a certain extent, stimulate strong and sustained antibody responses through a variety of ways, and induce cellular immune responses, making them an effective and safe vaccine delivery and adjuvant system to improve cancer immunotherapy.

## Summary and Perspectives

This review focuses on the anti-tumor activity obtained by polysaccharide compounds in stimulating or activating macrophages, dendritic cells, and NK cells to optimize the non-specific immunity, thereby regulating T cell function and, ultimately, enhancing the specific immunity. Most natural products have multiple “targets” that may affect different kinds of signaling pathways. Polysaccharide compounds can extensively regulate immune mechanisms through various pathways, such as TLR4, NF-κB, and notch pathways, which act on DCs, macrophages, and NK cells to achieve the balance and improvement of the immune microenvironment. The infiltration of activated immune effector cells in tumors is significantly related to the improvement of the prognosis of tumor diseases. Recent studies have found that dendritic cells and other immune cells are a key part of immunotherapy. The PD-L1 blockade can activate DC function, thereby generating a powerful anti-cancer T cell immunity (Mayoux et al., 2020), and that natural polysaccharides can also make efforts on it. A variety of immune cells stimulate the adaptive immunity, which increases the theoretical feasibility for subsequent studies of polysaccharides as immune adjuvants.

The components of plant polysaccharides and fungal polysaccharides have a lot in common, which makes them may have similar biological activities. However, there are great differences in structure between them, which determine the tendency of their biological functions to a certain extent. Plant polysaccharides are mainly pectin polysaccharides, whose immune-enhancing effects are mostly considered as 1→3, 6-branched galactose residues, the rhamnoides galacturonic acid and other structures and functional groups. While fungal polysaccharides are represented by β-glucan and its derivatives, they seem like to have better anti-tumor abilities and stronger immunomodulatory effects (Jiang et al., 2010). However, technical limitations such as separation and purification of polysaccharides prevent further structural and functional linkages, suggesting that we will need to link more common structures of different polysaccharides with their biological activities in the future. Plant and fungal polysaccharides had similar effects in activating macrophages and promoting their phagocytosis and cytokine release. However, for dendritic cells, plant polysaccharides, such as LBP (Zhu J. et al., 2007), have strong abilities to induce maturation, and are more likely to improve the tumor immune response. Regarding NK cells, generally speaking, fungal polysaccharides mostly promote their cytotoxicity and enhance anti-tumor activity, while plant polysaccharides promote the release of cytokines, such as interferon-γ and granzyme. In addition, different polysaccharides have preferences for the therapeutic effect of different cancers. Fungal polysaccharides may be more influential in hormone/endocrine-related tumors. *Ganoderma lucidum* polysaccharides have strong killing effect on liver cancer and colon cancer cell lines, but have no effect on osteosarcoma cells; compared with liver cancer, LNT has a better effect on breast cancer (Pang et al., 2018). Therefore, when we are faced with complex polysaccharides, we not only need to understand its basic structure, but also should compare and analyze it from multiple angles, combined with laboratory results, to comprehensively explore its possible ways of action.

Not only the extraction sources of polysaccharides, the structure, molecular weight of different polysaccharides, main chain and branched chain, functional group modification, spatial conformation and so on are also categories that we need to consider. Polysaccharides with special structure and functional group modification may have higher immunostimulatory activity. The polysaccharides with β-(1–3) bond and β-(1–6) branched chain on the main chain have anti-tumor activity, and their anti-tumor function may be obtained by regulating host immunity, such as (β1→4)-, (β1→3)-, and (β1→6)-D-glucans or (α1→3)-, (α1→4)-, and (α1→6)-d-glucans have better immune enhancement (Ferreira et al., 2015). In addition, the functional group modification mentioned in this review is also one of the leading factors in the biological activity of polysaccharides. we considered that triple-helixconformation can promote the release of TNF-α by macrophages and monocytes and enhance their immunostimulatory activity, and some experimental studies have shown that when breaking the triple helix conformation, the inhibitory activity of polysaccharides on sarcoma growth in mice is decreased. Single helix and polysaccharides without helix but with other residues may have immunostimulatory activity. Therefore, there is still a partially shared structure with the same biological activity between polysaccharides.

Although laboratory research has made some progress regarding the use of natural polysaccharides as immune adjuvants, there still are many challenges in the process of transferring them into clinical applications. The main problems, such as low oral availability and difficulty in targeting organs and tissues, need to be solved urgently. In response to these difficulties, researchers have made some remarks. First of all, different ways of administration can be chosen to improve their efficacy according to their biological characteristics. In the second place, different polysaccharides have complementary advantages, and two or more polysaccharides can be selected for synthesis [the combination of GLP and *Polyporus umbellatus* polysaccharides can enhance the innate immune function of mice (Gao et al., 2005a)] to improve their immune efficacy. Thirdly, combined with new materials, such as gold nanoparticles, polysaccharides can improve the bioavailability. Compared with free GLP, GLP-Au-induced CD80, CD86, CD40, and MHCII increased in a dose-dependent manner, and had a better immunostimulatory effect on DC maturation, with a statistically significant difference (Zhang et al., 2019). In addition, combining polysaccharides with liposomes (Huang Y. et al., 2016), or changing their properties to increase acetyl groups (Yue et al., 2020), or combining with proteins to form polypeptide compounds (Chen et al., 2009a) can improve their medicinal efficiency to varying degrees. Last but not least, it’s of vital for polysaccharides with the presence of triple-helix conformation of (β1→3)-D-glucans, function groups, branching degree of pectic polysaccharides, and Type II arabinogalactans to enhance immunostimulatory activity. We found that most plant polysaccharides have not been widely used in clinical preparations, while fungal polysaccharides are relatively widely used. Although the fundamental mechanism has not been fully explained, there are great differences in their clinical applications. We can reasonably infer that the dissimilarity may be due to the different molecular structure and bioavailability of their polysaccharides. In the future, we can organize the chain and conformation to form a database about polysaccharides, integrate its structural heterogeneity and function as much as possible, make it easy to obtain and analyze, and fully understand its biological activity.

In order to maximize the strengths and avoid weaknesses as much as possible, while fully exerting their potential, the efficiency of action needs to be improved. In short, the coordinated efforts of researchers and clinicians are essential to improve the drug defects of natural substances themselves. And we should also conduct further clinical trials to better develop the potential of natural phytochemicals for drug development.

## Glossary

ADCC: antibody-dependent cell-mediated cytotoxicity
ADCP: antibody-dependent cellular phagocytosis
APC: antigen presenting cell
APS: the *Astragalus* polysaccharide
AuNPs: gold nanoparticles
BRM: biological response regulators
cDC: conventional DC
CR3: complement receptor 3
CTL: cytotoxic T lymphocyte
CXCL: CXC chemokine ligand
DC: dendritic cell
DHP: Dendrobium huoshanense polysaccharides
DOP: Dendrobium officinale polysaccharides
ERK1/2: extracellular regulated protein kinases 1/2
GLP: *Ganoderma lucidum* polysaccharides
GM-CSF: granulocyte-macrophage colony-stimulating factor
GSP: *ginseng* polysaccharides
GSP-2: *Ganoderma sinense* polysaccharide
ICI: immune checkpoint inhibitor
IFN-γ: interferon-γ
IL-12: interleukin-12
iNOS: inducible nitric oxide synthase
LBP: *Lycium barbarum* polysaccharides
LPS: lipopolysaccharide
LNT: lentinan
MAPK: mitogen-activated protein kinase
MDSC: myeloid-derived suppressor cell
MDDC: bone marrow derived dendritic cell
MHC II: major histocompatibility complex II
M1: M1 macrophages
M2: M2 macrophages
NKs: natural killer cells
NO: nitric oxide
NPs: nanoparticles
NSCLC: non-small cell lung cancer
PBDC: peripheral blood dendritic cell
pDC: DC-specific precursor
PCPs: *Poria cocos* polysaccharides
PMs: peritoneal macrophages
PS-F2: G*anoderma formosanum* polysaccharides
PSG-1: *Ganoderma atrum* polysaccharide
RGP: *Rehmannia glutinosa* polysaccharide
ROS: reactive oxygen species
SMP: *Salvia miltiorrhiza* polysaccharide
SR: scavenger receptor
TAM: tumor-associated macrophages
Th1: T helper 1 cell
Th2: T helper 2 cell
TLR4: toll-like receptors 4
TME: tumor microenvironment
TNF-α: tumor necrosis factor-α
Treg: regulatory T cell
